# Plant Defense Stimulator Mediated Defense Activation Is Affected by Nitrate Fertilization and Developmental Stage in *Arabidopsis thaliana*

**DOI:** 10.3389/fpls.2020.00583

**Published:** 2020-05-26

**Authors:** Camille Verly, Atsin Claude Roméo Djoman, Martine Rigault, Frédéric Giraud, Loïc Rajjou, Marie-Emmanuelle Saint-Macary, Alia Dellagi

**Affiliations:** ^1^Institut Jean-Pierre Bourgin, INRAE, AgroParisTech, Université Paris-Saclay, Versailles, France; ^2^Staphyt-Service L&G/BIOTEAM, Martillac, France; ^3^Staphyt – BIOTEAM, Marsillargues, France

**Keywords:** defense (induced), elicitor, *Dickeya dadantii*, nitrate, developmental stage, Bion, Methyl-jasmonate, *Pseudomonas syringae* pv. *tomato*

## Abstract

Plant defense stimulators, used in crop protection, are an attractive option to reduce the use of conventional crop protection products and optimize biocontrol strategies. These products are able to activate plant defenses and thus limit infection by pathogens. However, the effectiveness of these plant defense stimulators remains erratic and is potentially dependent on many agronomic and environmental parameters still unknown or poorly controlled. The developmental stage of the plant as well as its fertilization, and essentially nitrogen nutrition, play major roles in defense establishment in the presence of pathogens or plant defense stimulators. The major nitrogen source used by plants is nitrate. In this study, we investigated the impact of *Arabidopsis thaliana* plant developmental stage and nitrate nutrition on its capacity to mount immune reactions in response to two plant defense stimulators triggering two major defense pathways, the salicylic acid and the jasmonic acid pathways. We show that optimal nitrate nutrition is needed for effective defense activation and protection against the pathogenic bacteria *Dickeya dadantii* and *Pseudomonas syringae* pv. *tomato*. Using an *npr1* defense signaling mutant, we showed that nitrate dependent protection against *D. dadantii* requires a functional *NPR1* gene. Our results indicate that the efficacy of plant defense stimulators is strongly affected by nitrate nutrition and the developmental stage. The nitrate dependent efficacy of plant defense stimulators is not only due to a metabolic effect but also invloves NPR1 mediated defense signaling. Plant defense stimulators may have opposite effects on plant resistance to a pathogen. Together, our results indicate that agronomic use of plant defense stimulators must be optimized according to nitrate fertilization and developmental stage.

## Introduction

As sessile organisms, plants are exposed to many biotic stresses such as pathogenic microorganisms and herbivores. They have developed the capacity to activate defenses in response to pathogen attacks thus leading to different degrees of resistance which may be effective at the site of infection or systemically (Jones and Dangl, 2006; Wirthmueller et al., 2013; Miller et al., 2017; Alhoraibi et al., 2019; Shine et al., 2019). Complex signaling networks are activated according to the type of invading organism (Bürger and Chory, 2019; Shine et al., 2019; Wang et al., 2020). Defense-related signaling responses involve phosphorylation events, ionic fluxes and accumulation of phytohormones leading to transcriptional activation of genes coding for the synthesis of antimicrobial compounds such as phytoalexins or pathogenesis related (PR) proteins (van Loon et al., 2006; Pieterse et al., 2014; Piasecka et al., 2015; Klessig et al., 2018; Wang et al., 2020).

Salicylic acid (SA) is one of the major hormones involved in plant immunity and was described as being mainly involved in plant protection against biotrophic or hemibiotrophic pathogens (Pieterse and Van Loon, 1999; Glazebrook, 2005; Zhang and Li, 2019). Defense genes activated by SA include *PR5*, encoding a thaumatin-like protein and *PR1*, encoding an antimicrobial protein with sterol binding and peptide signaling functions (Uknes et al., 1992; Chen et al., 2014; Gamir et al., 2017). Jasmonic acid (JA) and ethylene (ET) were reported to be involved in plant protection against necrotrophic pathogens (Pieterse and Van Loon, 1999; Glazebrook, 2005; Mengiste, 2012) and may be induced by non-pathogenic plant growth promoting rhizobacteria (PGPR) (Backer et al., 2018). JA/ET-dependent responses promote activities of peroxidases, polyphenol oxidases and lipoxygenases (Van Wees et al., 1999; Ruan et al., 2019). Genes encoding the defensin PDF1.2 and the lipoxygenase LOX2, are widely used as markers of the JA/ET defense pathway (Manners et al., 1998; Thomma et al., 1998; Yang et al., 2019). The JA pathway, with *LOX2* as a marker gene, is effective against insect pests (Glauser et al., 2009).

Several reports indicate the existence of cross-talks between those defense signaling pathways (Pieterse et al., 2011; Thaler et al., 2012; Vos et al., 2015; Bürger and Chory, 2019; Yang et al., 2019). An antagonism was generally described between SA dependent defenses and JA/ET dependent defenses (Koornneef and Pieterse, 2008; Leon-Reyes et al., 2010; Van der Does et al., 2013; Caarls et al., 2017; Li et al., 2019; Yang et al., 2019). The mechanisms underlying this antagonism imply transcriptional regulations involving transcription factors such as WRKY70 and ROXY19 (Li et al., 2004, 2019; Ndamukong et al., 2007; Caarls et al., 2015). Interestingly, synergism between SA and JA pathways was also described (Mur et al., 2006; Liu et al., 2016).

The NON-EXPRESSOR OF PATHOGENESIS-RELATED GENES 1 protein (NPR1) (Cao et al., 1997; Mou et al., 2003; Durrant and Dong, 2004) is a key defense regulator, known to be involved in both SA and JA/ET signaling pathways (Pieterse and Van Loon, 2004; Withers and Dong, 2017; Barker, 2018; Backer et al., 2019). Its role in SA defense signaling has been well-studied and described. NPR1 binds SA (Wu et al., 2012; Manohar et al., 2015) and is thought to be a co-receptor with two other proteins NPR3 and NPR4 which also bind SA and act as transcriptional repressors of the SA response (Kuai et al., 2015; Withers and Dong, 2016; Ding et al., 2018). Following SA perception, NPR1 binds to TGA transcription factors leading to the transcription of PR genes (Després et al., 2000; Kinkema et al., 2000; Zhou et al., 2000; Chern et al., 2001; Kuai et al., 2015). The mechanism by which NPR1 is involved in JA/ET defenses remains unclear. An *A. thaliana npr1* mutant plant fails to induce *PR* gene expression in response to SA, while *NPR1* overexpression leads to an up-regulation of the *PR* genes and enhanced disease resistance (Cao et al., 1998). NPR1 is also involved in the activation of JA/ET dependent defenses but probably via an alternate mechanism. An *A. thaliana npr1* mutant is unable to activate the JA/ET dependent defenses in response to PGPR (Koornneef and Pieterse, 2008; Nie et al., 2017). Overexpressing engineered forms of NPR1 retained in the plant cytosol results in the suppression of JA signaling (Spoel et al., 2003, 2007; Yuan et al., 2007) indicating that the antagonistic effect of SA over JA signaling requires cytosolic NPR1. Spoel et al. (2003) suggested a cytoplasmic role of NPR1 in the cross-talk between JA/ET and SA defense pathways.

Different environmental conditions may influence plant pathogen interactions such as the type of light (Kazan and Manners, 2011; De Wit et al., 2013; Janda et al., 2015; Mintoff et al., 2015) mineral nutrition (Poschenrieder et al., 2006; Walters et al., 2007; Fagard et al., 2014; Aznar et al., 2015) or water availability (Nejat and Mantri, 2017). The impact of fertilizers, in particular nitrogen fertilization, on plant-pathogen interactions is well-documented however, the underlying mechanisms remain unclear (Shaner and Finney, 1977; Eyles et al., 2007; Pato and Obeso, 2013; Veresoglou et al., 2013; Fagard et al., 2014; Mur et al., 2017; Sun et al., 2020).

Nitrogen is present in the form of ${\text{NO}}_{3}^{-}$, ${\text{NH}}_{4}^{+}$ or amino acids, the availability of which depends on physical factors such as pH and temperature. Plants adapted to acidic pH tend to take up ${\text{NH}}_{4}^{+}$ or amino-acids and plants adapted to higher pH and aerobic soils (which is the case of most arable lands) tend to prefer ${\text{NO}}_{3}^{-}$ (Masclaux-Daubresse et al., 2010). Nitrate is taken up at the root level by two different types of transport systems (Krapp et al., 2014; Wang et al., 2018): (1) a high affinity system involving the NRT1/NPF (nitrate transporter 1/peptide transporter family) family of transporters, (2) a low affinity system involving the NRT2 family of transporters. Following uptake, ${\text{NO}}_{3}^{-}$ is reduced to ${\text{NO}}_{2}^{-}$ by a cytosolic nitrate reductase, and then ${\text{NO}}_{2}^{-}$ is further reduced by a plastidial nitrite reductase into ${\text{NH}}_{4}^{+}$ (Masclaux-Daubresse et al., 2010). Ammonium is incorporated into amino acids in plastids via glutamate synthase (GS)/glutamine-2-oxoglutarate-aminotransferase (GOGAT) cycle (Masclaux-Daubresse et al., 2010; Krapp et al., 2014; Wang et al., 2018). Nitrogen fertilization has been a major factor in improving crop productivity in the last decades (Hirel et al., 2007; Wang et al., 2018) but may increase disease impact depending on the considered pathosystem (Fagard et al., 2014; Mur et al., 2017; Sun et al., 2020). A better understanding of the mechanisms by which nutrient elements influence plant defenses may be useful to improve cultural practices in order to optimize fertilization and reduce pesticide use thus decreasing the environmental impact of agriculture.

Emerging new plant protection strategies based on the exploitation of the capacity of plants to mount efficient immune responses are widely explored and are expected to allow the reduction of pesticide use (Ramamoorthy et al., 2001; Heil and Bostock, 2002; Bektas and Eulgem, 2015). These strategies rely on the use of plant defense stimulators which trigger plant defenses before or upon pathogen attack. Such plant defense stimulators include Bion^®^ which contains the bioactive molecule S-acibenzolar-S-Methyl activating the SA dependent defense pathway and is used in agriculture to protect tomato or apple against pathogens. However the effectiveness of plant defense stimulators in the field remains uncertain and may depend on different internal and/or external factors such as the plant developmental stage, temperature, drought, and/or mineral nutrition (Walters et al., 2007; Steimetz et al., 2012; Carella et al., 2015).

Our work addresses the combined impacts of the plant developmental stage and nitrogen nutrition on the efficiency of plant response to plant defense stimulators. We show that plant response to plant defense stimulators depends on both developmental stage and nitrogen nutrition with a stronger effect of nutrition.

## Materials and Methods

### Plant Material and Growth Conditions

Seeds of *Arabidopsis thaliana* Col-0 WT accession were obtained from Versailles Arabidopsis Stock Center (INRA Versailles France) and seeds of *npr1-1* mutant (N3726) in Col-0 WT background (Cao et al., 1997) were obtained from NASC^1^. Seeds were sown in unfertilized soil with different nitrate fertilization conditions 2, 10, and 26 mM of nitrate (Supplementary Table 1), in a growth chamber under the following conditions; 18 h of light 21°C, 6 h of dark 19°C, 70% relative humidity). Plants were grown until four different stages: Stage 1: plantlet (2 weeks after sowing (A.S.), Stage 2: vegetative stage (3 weeks A.S.), Stage 3: floral induction (4 weeks A.S.), Stage 4: flowering time (5 weeks A.S.).

### Nitrate Content Quantification

Leaves were harvested 48 h after treatment and immediately crushed in liquid nitrogen then stored at −80°C. Nitrate content is measured by a spectrophotometric method by comparison with a NaNO_3_ scale (Miranda et al., 2001). Ten milligrammes of frozen leaf powder were incubated in 300 μL of sterile distilled water during 20 min on ice. Samples were centrifuged at 15,000 rpm at 4°C during 20 min and supernatant was harvest twice. Ten microliters of supernatant were mixed with 90 μL of water and 100 μL of Miranda reagent (0.5M HCl, 0.25% Vanadium chloride, 0.005% *N*-1-naphtyethylendiamin, 0.1% Sulfanilamide) and incubated during 2 h at 60°C. Concentration of nitrate was then calculated based on a standard curve obtained with NaNO_3_ standard solutions by spectrophotometry at 540 nm. For each experiment 20 rosettes were used.

### Amino Acid Quantification

Amino acid content was measured by a spectrophotometric method adapted from Rosen (1957). Amino acids were extracted by vortexing 150 mg of frozen leaf powder with 1 mL of 2% 5-sulfosalicylic acid (w/v in water). Samples were then centrifuged at 13,000 rpm during 10 min and supernatant was harvested for the following steps. Fifty microliters of samples were mixed with 150 μL of water, 100 μL of cyanide acetate solution (0.2 mL of 10 mM KCN, 9.8 mL of 2.65M sodium acetate, 8% acetic acid pH 5.35) and 100 μl of ninhydrine solution (3% ninhydride in Ethylene glycol monomethylether) then incubated during 15 min at 100°C under fume hood, before adding 1 mL of 50% isopropanol. Samples were placed on ice to decrease temperature to room temperature. Two hundred microliters of samples were used to quantify amino acid content by comparison with standard solutions of L-glutamine by spectrophotometry at 570 nm. For each experiment 20 rosettes were used.

### RNA Extraction and cDNA Synthesis

RNA extraction and cDNA synthesis were performed as described in Aznar et al. (2014). For each experiment 6 to 10 plants were used.

### Quantitative PCR

Quantitative PCR reactions were carried out in 10 μL, with 5 μL of SybrGreen^®^ (Bio-Rad, Hercules, CA, United States) mix, 0.3 μM of each primer, and 2.5 μL of cDNA. Quantitative PCRs were carried out using a CFX-96 Real Time PCR system thermal cycler (Bio-Rad, Hercules, CA, United States). The raw data obtained were processed using the CFX manager software (Bio-Rad, Hercules, CA, United States). For each analysis, a cycle threshold (Ct) value was extracted and then transformed into Starting Quantity (SQ) values based on a standard curve equation. Consequently, for each condition, since PCRs were performed in triplicate, 3 SQ values were obtained for each sample and then averaged (geometric mean of SQ values). The geometric mean of the SQ values obtained for each gene of interest was then divided by the geometric mean of SQ values obtained for the reference gene. Normalized transcript level was then obtained and expressed as arbitrary units. Clathrin was used as a reference gene because it was stably expressed under the different stages and the different nutritional conditions. Experiments were performed three times with similar results. Representative data are shown.

### Plant Treatment With Plant Defense Stimulators

In all experiments, plants were kept under cover 24 h before plant defense stimulator treatment. Then, plants were sprayed with methyl-jasmonate (at 0.1 mM with 0.5% DMSO); the commercial plant defense stimulator Bion^®^ (at 0.015% in water w/v), or water. The Bion^®^ concentration was determined based on a calculation starting from the recommended dose for its agronomic use. The recommended Bion^®^, dose for tomato in the field (0.05 kg/ha) and was adapted in volume concentration (g/L) considering a field spray at 330 L/ha, this corresponds to a 0.015% in water w/v Bion^®^ solution. Spraying of the different elcicitors was performed separately to avoid cross contamination. Plants were kept under cover and grown in the same growth chamber. For each plant defense stimulator treatment, they were kept in separate boxes. Plants were then harvested 24 or 48 h following treatment, frozen in liquid nitrogen in order to extract RNA and amplify genes by qRT-PCR. For protection assays, plants were inoculated with the pathogenic bacteria as indicated below 48 h following plant defense stimulator treatments.

### Bacterial Strains and Inoculation Method

The *Dickeya dadantii* 3937 strain was obtained from our own collection. Bacteria were grown in Luria-Bertani medium. Forty-eight hours following water or plant defense stimulator treatment, bacterial inoculation was performed. For plant inoculation, a bacterial suspension at an OD600 of 0.1 (10^8^ C.F.U./mL) made up in 50 mM potassium phosphate buffer (pH 7) was used. Plants were covered during the whole assay to obtain saturating humidity and facilitate infection. To inoculate plants, a small hole was made with a needle in the leaf, and then, 5 μL of a bacterial suspension was deposited on the hole. In each experiment, 16 plants were inoculated for each condition and two leaves per plant were inoculated.

Disease severity levels were then scored 48 h post-inoculation (p.i) identified as the best timing for comparing disease severity (Rigault et al., 2017). The proportion of macerated surface in each inoculated leaf was calculated. The surface of the maceretad lesion and the surface of the corresponding leaves were measured using the open source software ImageJ https://imagej.nih.gov/ij/. This allowed calculating an average of lesion surfaces (in cm^2^) and an average of proportion of macerated surface for each leaf.

The *Pseudomonas syringae* pv. *tomato DC3000* bacterial strain was from our own stock. Forty-eight hours following plant defense stimulator treatment, plants were sprayed with a bacterial suspension at a concentration of 5 × 10^7^ cfu mL^–1^ in sterile water containing 0.01% Silwett. In planta bacterial populations were monitored 48 h after inoculation. Leaves were harvested then bacterial numbering was performed by tissue grinding followed by serial dilutions plated on King B medium with 60 μg/mL Rifampicin (King et al., 1954).

For each experiment 6 to 8 plants were used and 3 to 4 leaves were harvested or scored. This allowed us to analyze at least 20 leaves for each experiment.

### Statistical Analysis

All experiments were performed in three to four independent biological replicate. The size of all the samples is indicated in the figure legends. EXCEL-STAT plugin was used to perform statistical analysis on data.

## Results

### Plant Growth Modifications Under Different Nitrate Fertilization Conditions

**FIGURE 1:**
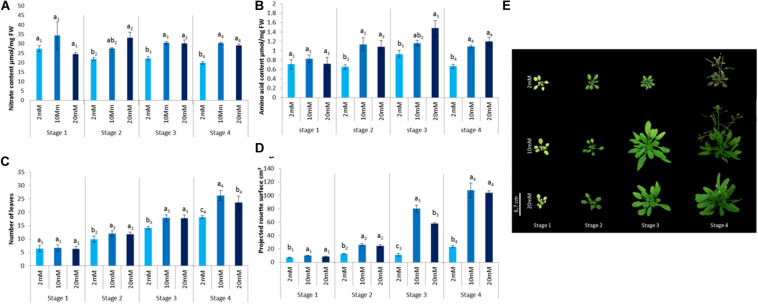
Impact of different nitrate fertilization levels on *Arabidopsis thaliana* physiological traits. Plants were cultivated until the indicated developmental stages (four stages) under the indicated nutritional conditions (2, 10, 26 mM). **(A)** Total nitrate content was quantified in healthy plants. **(B)** Total amino acid content was quantified. **(C)** Number of leaves per plant. **(D)** Projected rosette surface. **(E)** Picture of representative plants cultivated under indicated levels of nitrate at indicated developmental stages. *N* = 20. Error bars represent standard deviation. Letters indicate similarities or differences based on a *t*-test performed to compare samples of the same stage (*p* < 0.05). Experiments were performed three times with similar results. Representative data are shown.

The objective of this work was to evaluate both the effect of nitrogen nutrition and the developmental stage on defense activation by plant defense stimulators. For this purpose, different growth conditions in terms of nitrate supply and plant age were considered. In order to study the effect of developmental stages on defense activation in Arabidopsis, four developmental stages were considered based on the physiological steps representing four key phases in *A. thaliana* life cycle described in Boyes et al. (2001). In order to study the effect of nitrate nutrition, we chose to study three different levels: (1) limitation, (2) optimal fertilization, and (3) over-fertilization. In order to determine the nitrate concentrations required for these three physiological conditions, the following criteria were considered. A previous work on Arabidopsis nitrogen metabolism showed a differential accumulation of nitrogen related metabolites as well as enzymatic activities in plants cultivated under 2 and 10 mM nitrate (Lemaître et al., 2008). Plants grown under 10 mM nitrate displayed better growth than those grown under 2 mM nitrate (Loudet et al., 2003; Lemaître et al., 2008). Based on these data, the nitrate limitation nutrition used in the present study, was 2 mM nitrate. For the optimal growth conditions, in the present study, we used the 10 mM nitrate. Although, under agronomic conditions over-fertilization occurs quite regularly, it is unclear how over fertilization can affect plant defenses. To address this question, we determined a nitrate level higher than 10 mM, resulting in a reduced plant growth without being lethal or affecting too much plant development. For this purpose, plants grown under 20, 26, and 50 mM nitrate were tested. The 26 mM nitrate concentration slightly affected Arabidopsis growth (Figure 1) without being lethal; while 20 mM did not significantly affect plant growth and 50 mM was toxic (data not shown). To confirm that the three nitrate fertilization conditions have different impacts on plant physiology and/or development under the three growth conditions, we determined the impact of nitrate nutrition on some physiological and/or metabolic traits (Figure 1). Nitrate and amino acid contents were monitored. Plant growth was quantified via the number of leaves per plant and the projected rosette surface. Plants grown under limiting nitrate levels (2 mM) displayed lower nitrate, amino acid and reduced leaf number and rosette surface at stage 2 to stage 4. This indicates that the lower nitrate 2 mM supply has an impact on plant nitrogen metabolism that can be observed starting from the stage 2. Although growing plants under 26 mM nitrate did not result in an increase in nitrate or amino acid content, the number of leaves (stage 4) and the projected rosette area (stage 3) were affected compared to those grown under 10 mM nitrate (Figure 1). This indicates that 26 mM nitrate supply has a negative impact on plant development which can be observed starting from stage 3.

Together these data indicate that the three nutritional regimes impacted differently the plant physiological status and nitrogen metabolism. The 2 mM nitrate nutritional condition is limiting and 26 mM nitrate nutritional condition corresponds to an over-fertilization, while 10 mM nitrate corresponds to an optimal nitrate supply.

### Plant Developmental Stage Affects the Capacity to Activate Defense Gene Expression in Response to Plant Defense Stimulators

**FIGURE 2:**
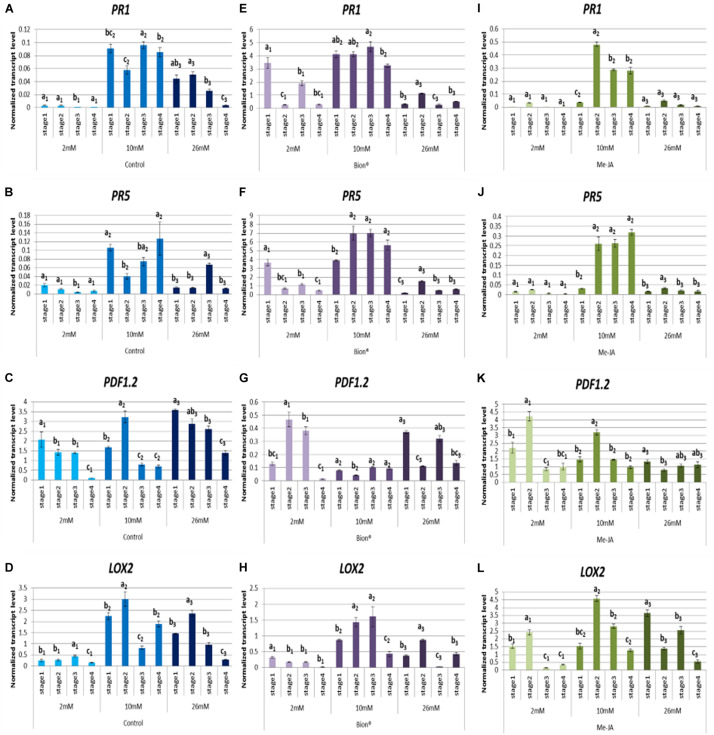
Impact of developmental stage on defense gene expression 48 h after plant defense stimulator treatment. Plants were cultivated until the indicated developmental stages (four stages) under the indicated nutritional conditions (2, 10, 26 mM). They were treated with Bion^®^, MeJA or water as a control then harvested 48 h after treatment, 6 to 10 plants were harvested for each treatment in each experiment. Defense gene expression was monitored by q-RT-PCR. Data represent normalized transcript levels using Clathrin as a reference gene. **(A–D)** Indicate defense gene expression following water treatment, **(E–H)** Indicate defense gene expression following Bion^®^ treatment; **(I–L)** Indicate defense gene expression following MeJA treatment. Experiments were performed 3 to 4 times with similar results. Representative data are shown. Error bars represent standard error. Different letters represent statistically significant differences between plant defense stimulator treatments under the same nutritional condition (*p* < 0.05 as calculated by *t*-test).

During the different phases of plant development, important metabolic and transcriptomic changes occur which may affect basal defenses and activation of immune responses. These modifications could account for the variability of plant defense stimulator activity under agronomic conditions. To address this point, Arabidopsis plants were grown until the four developmental stages considered as key steps in Arabidopsis life cycle (Boyes et al., 2001). Three nitrate fertilization levels were applied (2, 10, and 26 mM). Plants were treated with either MeJA, which is known to activate the JA/ET defense pathway, or with Bion^®^, which is known to activate the SA pathway. Expression profiles of two marker genes of the SA pathway (*PR1* and *PR5*) and two marker genes of the JA/ET pathway (*LOX2* and *PDF1.2*) were monitored by qRT-PCR. To determine the time post-plant defense stimulator treatment the most relevant to monitor defense gene expression, plants were collected 24 and 48 h following Bion^®^ treatment at the four developmental stages and under 2 and 10 mM nitrate nutritional conditions. These experiments showed that the highest level of gene expression was reached 48 h after treatment (Supplementary Figure 1). Thus, the rest of the experiments were performed by analyzing gene expression 48 h after plant defense stimulator treatment. In order to determine the effect of the plant developmental stage on defense gene expression, normalized transcript levels were compared under each treatment and each nitrate nutrition separately (Figure 2). Figure 2 shows that plant developmental stage significantly affects most of the defense responses. Basal defenses are significantly affected by developmental stage as indicated by the expression profiles of the four marker genes in control pants (Figures 2A–D). The effect of developmental stage on defense activation by plant defense stimulators is significant under the three nutritional conditions. Interestingly, nitrate supply impacts the effect of stage on defense activation (Figure 2 and Supplementary Figures 2, 3). For example, at 2 mM nitrate supply, *PR1* and *PR5* transcript levels in response to Bion^®^ are significantly reduced at stage 4 compared to other stages, while at 10 mM nitrate supply *PR1* and *PR5* transcript levels are globally high at all stages. Although the transcript level of *PR1* and *PR5* are low following MeJA treatment, they accumulate differentially depending on the developmental stage when plants are grown under 10 mM nitrate. There is no stage effect on *PR1* and *PR5* transcript levels in response to MeJA under limiting nitrate or over-fertilization. The transcript levels of the two JA/ET markers genes *PDF1.2* and *LOX2* are affected by the developmental stage whatever the nitrate nutrition. These two markers are more highly expressed at stage 2 following MeJA treatment under 2 and 10 mM nitrate, compared to the other stages. Together these data indicate that depending on the nutrition, developmental stage plays crucial role in the plant defense system.

### Nitrate Fertilization Affects Plant Capacity to Activate Defense Gene Expression in Response to Plant Defense Stimulators

**FIGURE 3:**
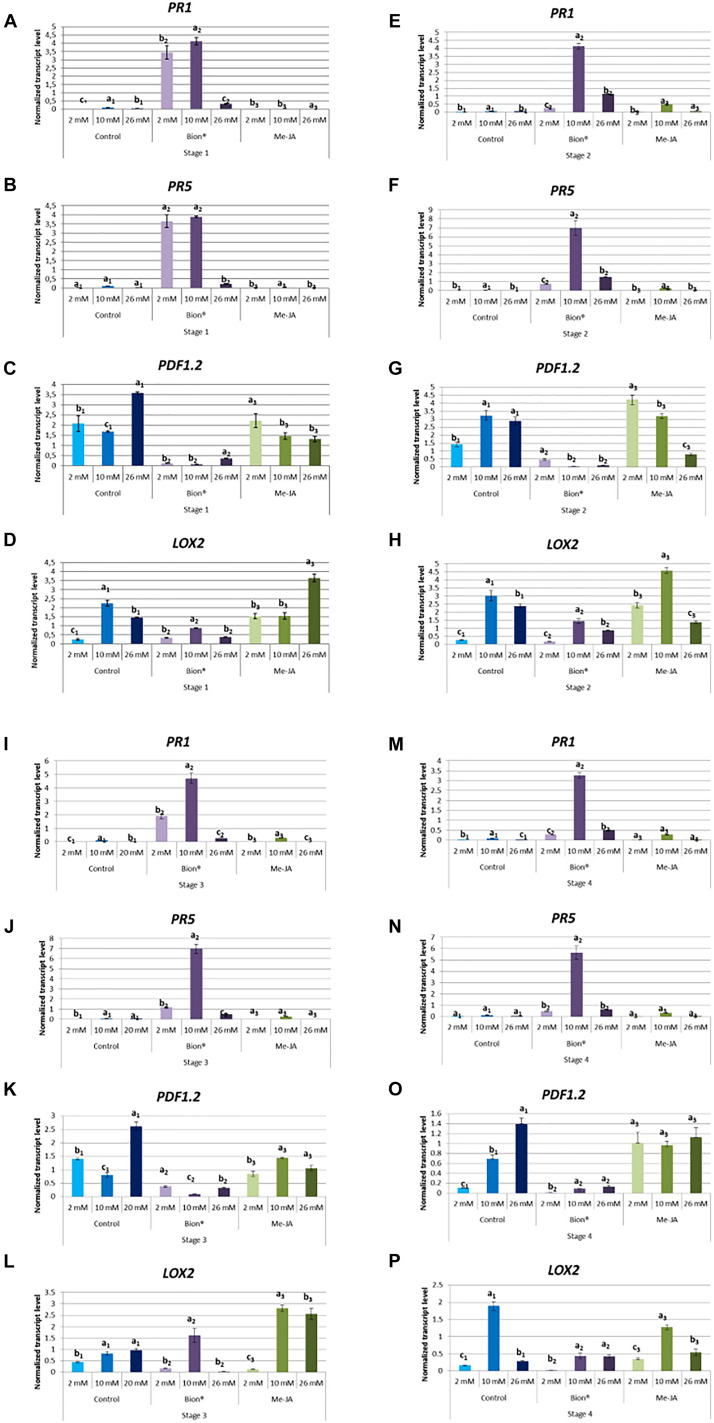
Impact of nitrate nutrition on defense gene expression 48 h after plant defense stimulator treatment. Plants were cultivated until the indicated developmental stages (four stages) under the indicated nutritional conditions (2, 10, 26 mM). They were treated with Bion^®^, MeJA or water as a control then harvested 48 h after treatment, 6 to 10 plants were harvested for each treatment in each experiment. Defense gene expression was monitored by q-RT-PCR. Data represent normalized transcript levels using Clathrin as a reference gene. **(A–D)** Indicate defense gene expression at stage 1; **(E–H)** Indicate defense gene expression at stage 2; **(I–L)** Indicate defense gene expression at stage 3; **(M–P)** Indicate defense gene expression at stage 4. Experiments were performed 3 to 4 times with similar results. Representative data are shown. Error bars represent standard error. Different letters represent statistically significant differences between plant defense stimulator treatments under the same nutritional condition (*p* < 0.05 as calculated by *t*-test).

To determine whether nitrate fertilization affects defense activation, plants were treated with either MeJA or with Bion^®^ and expression profiles of two maker genes of the SA pathway (*PR1* and *PR5*) and two marker genes of the JA/ET pathway (*LOX2* and *PDF1.2*) were monitored by qRT-PCR 48 h after plant defense stimulator treatment (Figure 3). Figure 3 shows that nitrate nutrition significantly affects most of the defense responses. Basal defenses are in most cases significantly affected by nitrate as indicated by the expression profiles of the four marker genes in control pants (Figure 3). At all developmental stages, nitrate supply significantly affected the expression of the SA markers following Bion^®^ treatment and the highest expression of these markers was obtained under 10 mM nitrate. Nitrate supply significantly affected the expression levels of the JA/ET defense markers following MeJA treatment. Interestingly, Bion^®^ treatment results in the down regulation of PDF1.2 and LOX2 genes under all nitrate treatments (Supplementary Figures 2, 3).

Together these data indicate that plant defense stimulator mediated defense gene activation depends both on the stage and on nitrate nutritional condition.

### Nitrate Supply Affects Plant Defense Stimulator Mediated Protection Against *Dickeya dadantii* and *Pseudomonas syringae* pv. *tomato*

The enterobacterium *D. dadantii* is a necrotrophic plant pathogen able to infect *A. thaliana* plants causing maceration symptoms as a results of the secretion of large amounts of plant cell degrading enzymes (Reverchon and Nasser, 2013). The model plant *A. thaliana*, in turn, activates different defenses to limit infection, such as the JA/ET defense pathway and the accumulation of reactive oxygen species (Fagard et al., 2007). The Gram negative bacterium *P. syringae* pv. *tomato* is a model plant pathogen (Xin and He, 2013). The SA signaling pathway is known to promote Arabidopsis defense against *P. syringae* pv. *tomato* and it is commonly used to monitor plant defense stimulator activities (McCann et al., 2012; Rufián et al., 2019). To know whether nitrate fertilization may influence the plant defense stimulators mediated protection, we decided to use plants at vegetative stage (stage 2). Indeed, at stage 2, differential expression profiles of defense genes were observed in response to Bion^®^ and MeJA allowing a better interpretation of the putative connection between protection and defenses. In addition, this stage is commonly used in most studies, allowing a better interpretation of the data compared to the literature (Rufián et al., 2019). Two days after plant defense stimulator or water treatment, plants were inoculated with *P. syringae* pv. *tomato* or *D. dadantii*.

**FIGURE 4:**
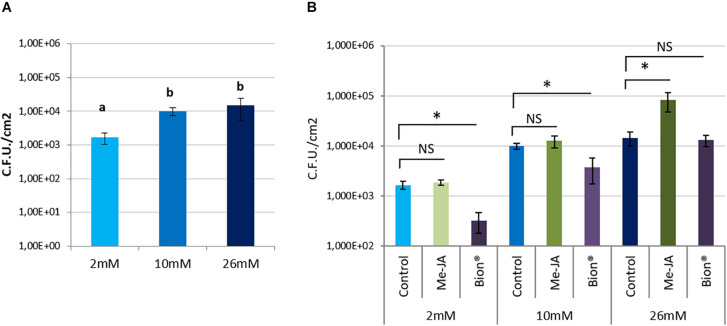
Impact of nitrate fertilization on protection against the pathogenic bacterium *Pseudomonas syringae* pv. *tomato* after plant defense stimulator application. Plants were cultivated until developmental stage 2 (rosette) under the indicated nitrate nutritional conditions (2, 10, 26 mM). They were treated with Bion^®^, MeJA or water as a control, then inoculated with *P. syringae* pv. *tomato* 48 h after plant defense stimulator treatment. Bacterial populations were monitored. Error bar represent standard deviation. *N* = 20 leaves. Different letters **(A)** or stars **(B)** represent statistically significant differences between control and plant defense stimulator treatments under the same nutritional conditions, NS represent “No Significant” differences (*p* < 0.05 as calculated by *t*-test). Experiments were performed three times with similar results. Representative data are shown.

To know whether nitrate supply affects *A. thaliana* defenses against *P. syringae* pv. *tomato*, bacterial populations were monitored in control plants and compared with Bion^®^ or MeJA treated plants under the three nitrate supply conditions. Symptoms caused by *P. syringae* pv. *tomato* can be seen in Supplementary Figure 5. Nitrate limitation (2 mM) resulted in reduced plant susceptibility to *P. syringae* pv. *tomato* in control plants, indicating that basal defenses against *P. syringae* pv. *tomato* are more efficient in nitrate starved plants than in optimally or over-fertilized plant (Figure 4A). Bion^®^ treatment resulted in plant protection under 2 and 10 mM nitrate supply but was inefficient on over-fertilized plants. Interestingly, over-fertilization resulted in enhanced plant susceptibility to *P*. *syringae* pv. *tomato* following MeJA treatment (Figure 4B).

**FIGURE 5:**
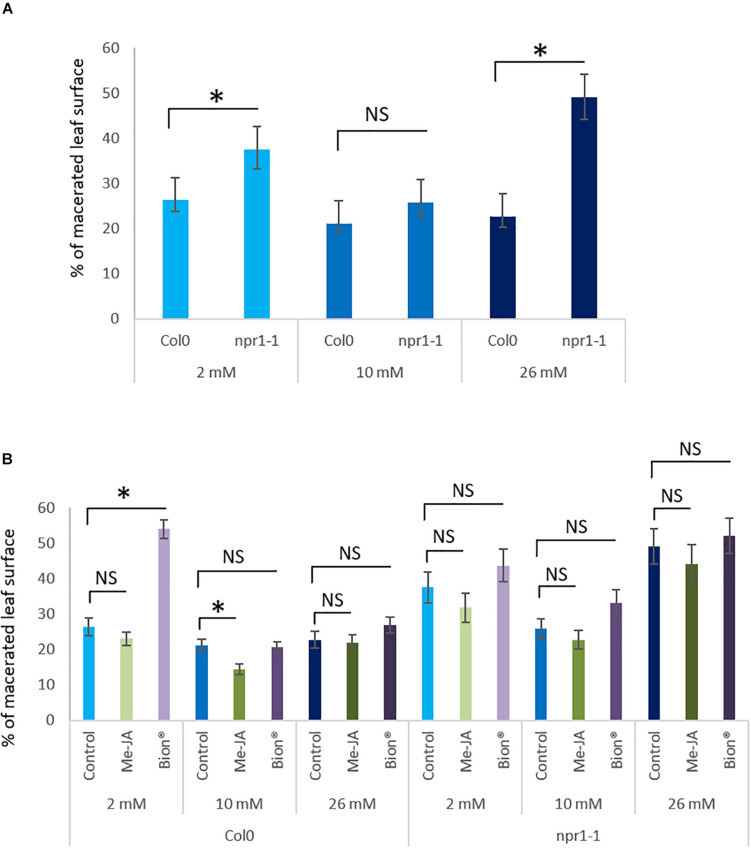
Impact of nitrate fertilization on protection against the pathogenic bacterium *D. dadantii* after plant defense stimulator application on an *npr1* mutant. Plants were cultivated until developmental stage 2 (rosette) under the indicated nitrate nutritional conditions (2, 10, or 26 mM). They were treated with Bion^®^, MeJA or water as a control, then inoculated with *D. dadantii* 48 h after plant defense stimulator treatment. Symptoms were scored 48 h after inoculation. Percentage of macerated leaf surface are represented. Error bars represent standard error. *N* = 20 leaves. Stars represent statistically significant differences between *npr1-1* mutant plants and wild type Col-0 WT **(A)** and between control and MeJA and Bion^®^, treatment **(B)** for each level of fertilization. NS represent “No Significant” differences (*p* < 0.05 as calculated by *t*-test). Experiments were performed three times with similar results. Representative data are shown.

To know whether nitrate supply affects basal *A. thaliana* susceptibility to the *D. dadantii*, symptom severity on water treated control plants were compared under the three nitrate supply conditions. The level of nitrate fertilization had no effect on the proportion of macerated leaf surface in non-elicited plants (Figure 5A). Following MeJA treatment, the proportion of leaf macerated surface was decreased under optimal nitrate supply (10 mM), but no effect of MeJA was observed when plants were under-fertilized or over-fertilized (Figures 3B,D and Supplementary Figure 4). These data indicate that MeJA is efficient to protect *A. thaliana* against *D. dadantii* under optimal nitrate supply only. The proportion of leaf macerated surface was increased following Bion^®^ treatment under 2 mM nitrate fertilization but unaffected by Bion^®^ under 10 or 26 mM nitrate nutritional conditions.

These data indicate that the level of nitrate fertilization influences the capacity of the plant to activate efficient defenses following plant defense stimulator treatments against necrotrophic pathogens such as *D. dadantii* and hemibiotrophic pathogens such as *P. syringae* pv. *tomato*.

### *NPR1* Gene Is Involved in Nitrate Dependent Plant Defense Stimulator Mediated Defense Responses

As a key player in plant immunity, NPR1 was shown to be involved in both SA and JA/ET signaling pathways (Spoel et al., 2003; Pieterse and Van Loon, 2004; Mao et al., 2007). We investigated whether nitrate supply affected susceptibility of the *npr1-1* mutant to *D. dadantii*. For this purpose, symptom severity on Col-0 WT plants were compared to that of *npr1-1* mutant in water treated control plants under the three nitrate supply conditions. Over-fertilization and under-fertilization increased *npr1-1* mutant susceptibility compared to WT (Figure 5A) indicating that *NPR1* is required for the plant basal defense against *D. dadantii*. Plant defense stimulators activity was monitored in the *npr1-1* mutant background to know whether *NPR1* was involved in the plant response to plant defense stimulators under the different nitrate nutritional conditions. Interestingly, both plant defense stimulator treatments failed to show any effect on plant disease severity in the *npr1-1* mutant whatever the level of fertilization used (Figure 5B). Thus, while we observed on WT plants an impact of Bion^®^ and MeJA on plant protection against *D. dadantii*, no effect was observed on the *npr1-1* mutant plants.

These data suggest that nitrate dependent defense activation by plant defense stimulators requires a functional plant defense signaling machinery which likely involves *NPR1*.

**FIGURE 6:**
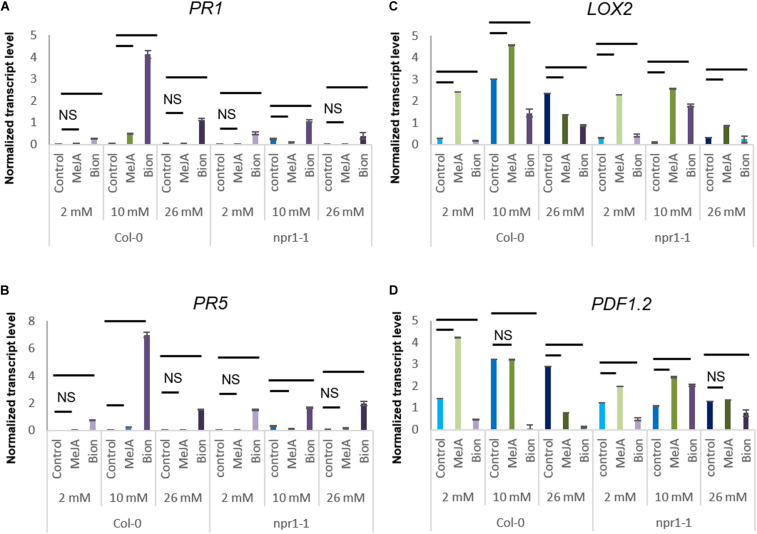
Impact of nitrate nutrition on defense gene expression 48 h after elicitor treatment. Plants were cultivated until the developmental stage 2 under the indicated nutritional conditions (2, 10, 26 mM nitrate). They were treated with Bion^®^, MeJA or water as a control then harvested 48 h after treatment, 6 to 10 plants were harvested for each treatment in each experiment. Transcript levels of indictade genes, *PR1* **(A)**, *PR5* **(B)**, *LOX2* **(C)**, and *PDF1.2* **(D)** was monitored by qRT-PCR. Data represent normalized transcript levels using Clathrin as a reference gene. Bars indicate comparisons between control and plant defense stimulator treatments under the same nutritional condition. When only a bar is visible, the difference is significant (*p* < 0.05 as calculated by *t*-test). NS: statistically Non-Significant differences between control and plant defense stimulator treatments under the same nutritional condition.

To investigate whether the role of *NPR1* in protection against *D. dadantii* following MeJA treatment and increased susceptibility to *D. dadantii* following Bion^®^ treatment would involve the SA and/or the ET/JA, defense gene expression was monitored in the *npr1-1* mutant and compared to their expression in the Col-0 WT. For this purpose, *npr1-1* mutant plants were grown until stage 2 under 2, 10, or 26 mM, treated with water (control), Bion^®^ or MeJA and transcript levels of defense genes were monitored by qRT-PCR. Figures 6A,B indicates that, as expected, the expression level of the two SA markers genes *PR1* and *PR5* is drastically reduced in the *npr1-1* mutant compared to Col-0 WT. The expression profiles of *LOX2* was similar in Col-0 WT and *npr1-1* under the nutrition conditions of 2 and 10 mM nitrate. Interestingly, the *LOX2* transcript level was globally lower in *npr1-1* plants under 26 mM nitrate compared to Col-0 WT plants (Figure 6C) which could explain the enhanced susceptibility of to *D. dadantii* in *npr1-1* naive plants compared to Col-0 WT naïve plants (Figure 5A). The increased susceptibility of Col-0 WT plants to *D. dadantii* under 2 mM nitrate following Bion^®^ treatment (Figure 5B) is consistent with a down-regulation of *PDF1.2* and *LOX2* expression in Col-0 following Bion^®^ treatment under 2 mM nitrate (Figures 6C,D). Although this increased susceptibility is abolished in the *npr1-1* mutant under these conditions, the down-regulation of *PDF1.2* and *LOX2* is still observed.

The protection of Col-0 WT plants agianst *D. dadantii* under 10 mM nitrate following MeJA treatment (Figure 5B) is consistent with an up-regulation of *LOX2* expression in Col-0 following MeJA treatment under 10 mM nitrate (Figure 6C). Although this protection is abolished in the *npr1-1* mutant, under these conditions, the up-regulation of *LOX2* is still observed.

Taken together, these data indicate that *NPR1* plays an important role in modulating Arabidopsis defenses depending on nitrate supply.

## Discussion

Most of the plant protection treatments directly target invading pathogens. In general, this kind of practices has proven to cause pathogen resistance toward pesticides, thus reducing their efficiency (Hahn, 2014). In addition, pesticide use has detrimental effects on animal health and environment. It is nowadays obvious that alternate and sustainable plant protection strategies are needed to avoid the detrimental effects of pesticide and reduce pathogen adaptation (Pretty, 2018). The use of plant defense stimulators is one of the proposed alternate crop protection strategies which is being investigated by scientists and farmers because they don’t directly target the pathogen and they provide a wide protection range. However, plant defense stimulators efficiency is controversial. While they can protect plants from pathogen infections under controlled conditions, their efficiency in the field is often unstable (Bektas and Eulgem, 2015).

In this work, we investigated the possibility that plant responses to plant defense stimulators could be affected by the developmental stage and nitrogen nutrition. The objective was to determine whether by adjusting fertilization and targeting specific developmental stages, plant defense stimulators use could be optimized.

Plant intrinsic susceptibility to pathogens depends on plant developmental stage and nitrogen status. For instance, the bacterial fire blight causing pathogen *Erwinia amylovora* preferentially infects growing tissues and apple flowers (Malnoy et al., 2011). Conversely, senescence can be a factor which favors necrotrophic pathogen infection while it prevents biotrophic pathogen infections (Häffner et al., 2015). On the other hand, plant intrinsic susceptibility to pathogens can vary depending on the nitrogen fertilization. Complex interactions have been described concerning the connection between plant nitrogen status and tolerance to pathogens (Fagard et al., 2014; Mur et al., 2017; Sun et al., 2020). For instance, nitrate fertilization increases tomato tolerance to the fungal necrotroph *Botrytis cinerea* (Lecompte et al., 2010); while it increases the susceptibility of *A. thaliana* to this fungus (Fagard et al., 2014). Nitrogen fertilization has an impact on defense activation (Kruse et al., 2007; Kutyniok and Müller, 2013; Mur et al., 2017; Zarattini et al., 2017; Farjad et al., 2018) as well as on pathogen virulence factors (Van den Ackerveken et al., 1994; Snoeijers et al., 2000; Robert et al., 2004). Thus, the impact of nitrogen status on plant tolerance/susceptibility does not exclusively depend on nutritional availability to pathogen, but involves complex mechanisms.

The above-cited reports describe the impact of the developmental stage and nitrogen fertilization on plant intrinsic susceptibility to pathogens. However, very few data are available about the impact of the developmental stage and nitrogen fertilization on plant defense stimulator mediated defense activation. Our data show that the plant defense responses to two plant defense stimulators, which trigger two major defense signaling pathways are affected by both the developmental stage and nitrate nutrition in *A. thaliana*. Activation of SA pathway by Bion^®^ was dependent of both the nitrate supply and the developmental stage (Figures 2, 3), indicating that the fertilization and physiological stage parameters should be considered when using Bion^®^ as an plant defense stimulator. Optimal nitrate nutritional conditions were the most favorable conditions for SA defense activation by Bion^®^. Dietrich et al. (2004) showed that nitrogen limitation resulted in reduced defense induction by Bion^®^. In a transcriptomic approach to characterize the combined effect of pathogen and nitrogen deficiency, Farjad et al. (2018) showed that the upregulation of a set of defense related genes was higher under nitrogen limitation. Thus, depending on the biotic stress and the defense pathway considered, nitrogen deficiency can differentially affect immune responses. These studies did not investigate over-fertilization conditions.

Regulation of *LOX2* and *PDF1* transcript levels following MeJA treatment was strongly affected by nitrate supply and developmental stage (Figures 2, 3). Up-regulation was not observed in all cases and it was surprising to observe repression of these markers following MeJA treatment in some cases (Supplementary Figure 3). Both ET and JA play important roles in plant development (Huang et al., 2017; Dubois et al., 2018). Thus, the differential expression observed between stage 1 and 4 may be in part due to their accumulation level during these key developmental phases. Interestingly, an up-regulation of *PR1* and *PR5* was observed following MeJA treatment although to a lower level than those observed following Bion^®^ treatment. This dual activation of SA and JA pathways was also recently described in the context of plant resistance mediated by a specific resistance gene in Arabidopsis (Liu et al., 2016) and may be more common than usually assumed. The ET/JA pathway is recruited during induced systemic resistance (ISR) triggered by PGPR (Backer et al., 2018). It would be interesting to determine whether ISR is affected by plant developmental stage and nutrition.

The impact of nitrate supply on effective protection of MeJA and Bion^®^ against two bacterial pathogens with different lifestyles was investigated. Bion^®^ conferred protection against the hemibiotrohoic bacterium *P. syringae* pv. *tomato* under low and optimal nutrition but failed to protect under high nitrate. Conversely, MeJA treatment resulted in an increased plant susceptibility. Protection was conferred by MeJA against *D. dadantii* when plants were cultivated under optimal nitrate nutritional conditions with 10 mM nitrate, while no protection was observed under low or over-fertilization conditions. This optimal protection is not perfectly correlated with transcriptional activation of *PDF1.2* and *LOX2*, indicating that these two defense markers do not fully explain the protection at optimal nitrate nutritional conditions. Interestingly, *LOX2* expression, is up-regulated at stage 2 and under optimal nitrate nutrition which correspond to the conditions where MeJA protects plants against *D. dadantii* (Supplementary Figure 3) indicating that the JA pathway could be acting here via ISR. These data are consistent with the fact that JA is involved in *A. thaliana* defense against *D. dadantii* (Fagard et al., 2007; Reverchon and Nasser, 2013). While MeJA conferred protection against *D. dadantii*, Bion^®^ treatment resulted in an increased susceptibility. This increased susceptibility could be explained by the repression of ET/JA defenses we observed following Bion^®^ treatment which activates the SA pathway (Supplementary Figure 2). An antagonistic effect of the SA pathway over the ET/JA pathway was previously described (Koornneef and Pieterse, 2008; Leon-Reyes et al., 2010; Van der Does et al., 2013; Caarls et al., 2017; Li et al., 2019; Yang et al., 2019). Care must be taken when fighting diverse bioagressors in the field since plant defense stimulators can have opposite effects.

In order to determine the defense signaling contribution in the plant protection mediated by MeJA against *D. dadantii*, the *npr1-1* mutant was used because this mutant was described as being affected in both the SA and the ET/JA defense responses (Pieterse and Van Loon, 2004; Withers and Dong, 2017; Barker, 2018; Backer et al., 2019). The enhanced susceptibility of the *npr1-1* mutant could be surprising since this gene is commonly known to activate SA response which is effective against biotrophs. In the *npr1-1* mutant, one could expect the increase in JA signaling leading to enhanced resistance to the necrotroph *D. dadantii*. However, several examples show that NPR1 overexpression leads to tolerance to necrotrophic pathogens (Wally et al., 2009; Ali et al., 2017). Our data show that MeJA mediated *A. thaliana* protection against *D. dadantii* requires *NPR1*. Similarly, Bion^®^ mediated plant increased susceptibility to *D. dadantii* is abolished in the *npr1-1* mutant. It is intriguing that both increased and decreased protection involve *NPR1*. To tackle this issue, the role of the SA and ET/JA defense signaling pathways in the defense modulation by NPR1 was investigated by monitoring the expression of defense gene markers of these pathways in the *npr1-*1 mutant under the different nutritional conditions and following plant defense stimulator treatments. Interestingly, *LOX2* expression seems to both depend on *NPR1* and nitrate supply. Indeed, *LOX2* expression was strongly reduced in *npr1-1* mutant plants under 26 mM nitrate correlating with enhanced susceptibility to *D. dadantii*. Our data illustrate the complexity by which NPR1 is involved in the balance between the SA and the ET/JA signaling pathways that remains to be further investigated (Pieterse et al., 2011; Li et al., 2019).

The role of NPR1 in the nitrate dependent defense modulation by plant defense stimulators suggests a role of nitrate nutrition on defense signaling mechanisms. One possible mechanism by which nitrate nutrition can interact with defense signaling is via NO accumulation which can be a byproduct of nitrate reductase. *A. thaliana* plants fertilized with nitrate accumulated higher levels of NO than ammonium fed plants suggesting an involvement of NO in the higher tolerance of nitrate fertilized plants to the pathogenic bacterium *P. syringae* (Gupta et al., 2012). The role of NO could be related to the activity of the NPR1 protein which is known to be S-nitrosylated (Tada et al., 2008; Withers and Dong, 2017).

These data support the idea that the impact of nitrate nutrition in plant immunity is complex and probably involves interactions between defense signaling pathways and metabolic pathways.

Our data could be useful to the design of performant agronomic practices by choosing and adapting the best fitted conditions for the use of plant defense stimulators taking into account the stage of development and the nitrogen status.

## Data Availability Statement

All datasets generated for this study are included in the article/Supplementary Material.

## Author Contributions

AD and M-ES-M conceived conceptually the work. CV and AD wrote the manuscript. CV, ACRD, and MR performed the experiences. AD, M-ES-M, LR, and FG supervised the project.

## Conflict of Interest

ACRD, CV, MM, and FG were employed by the company STAPHYT. The remaining authors declare that the research was conducted in the absence of any commercial or financial relationships that could be construed as a potential conflict of interest.

## Footnotes

1 http://www.arabidopsis.info
