# Supplementary material for: Plant Defense Stimulator Mediated Defense Activation Is Affected by Nitrate Fertilization and Developmental Stage in Arabidopsis thaliana
Source: Front Plant Sci. 2020 May 26;11:583. doi: 10.3389/fpls.2020.00583 (PMC7264385; doi:10.3389/fpls.2020.00583)
Supplement: Supplementary file 1 [file Data_Sheet_1.pdf]

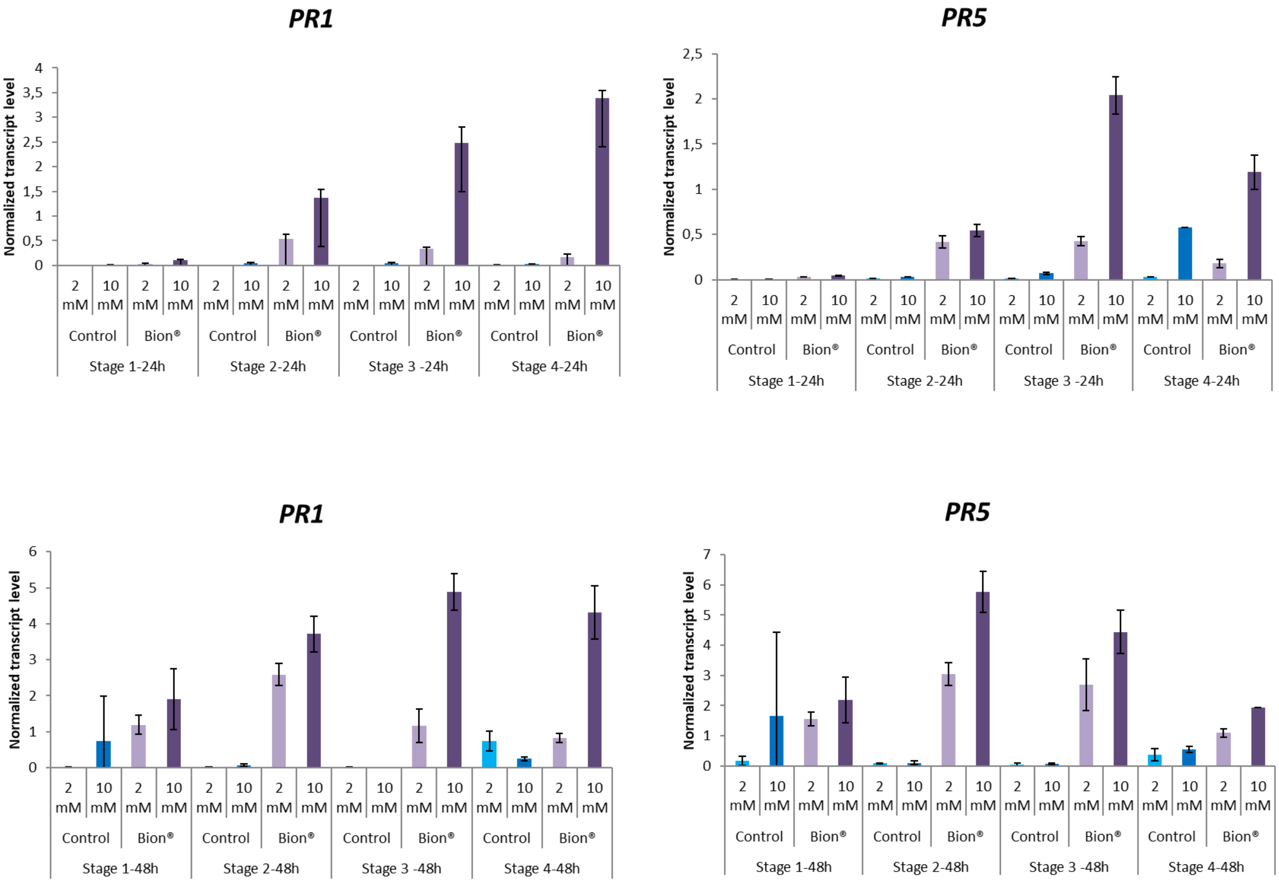

Supplementary Figure 1: Defense gene expression at 24 h and 48 h following Bion treatment. Plants were cultivated until the indicated developmental stages (4 stages) under the indicated nitrate nutritional conditions. They were treated with Bion or water as a control then harvested 24h or 48 h after treatment as indicated. Defense gene expression was monitored by q-RT-PCR. These data show that defense expression is globally higher 48 hours following elicitor treatment. Data represent normalized transcript levels using Clathrin as a reference gene. Experiments were performed 3 times with similar results. Representative data are shown. Error bars represent standard error.

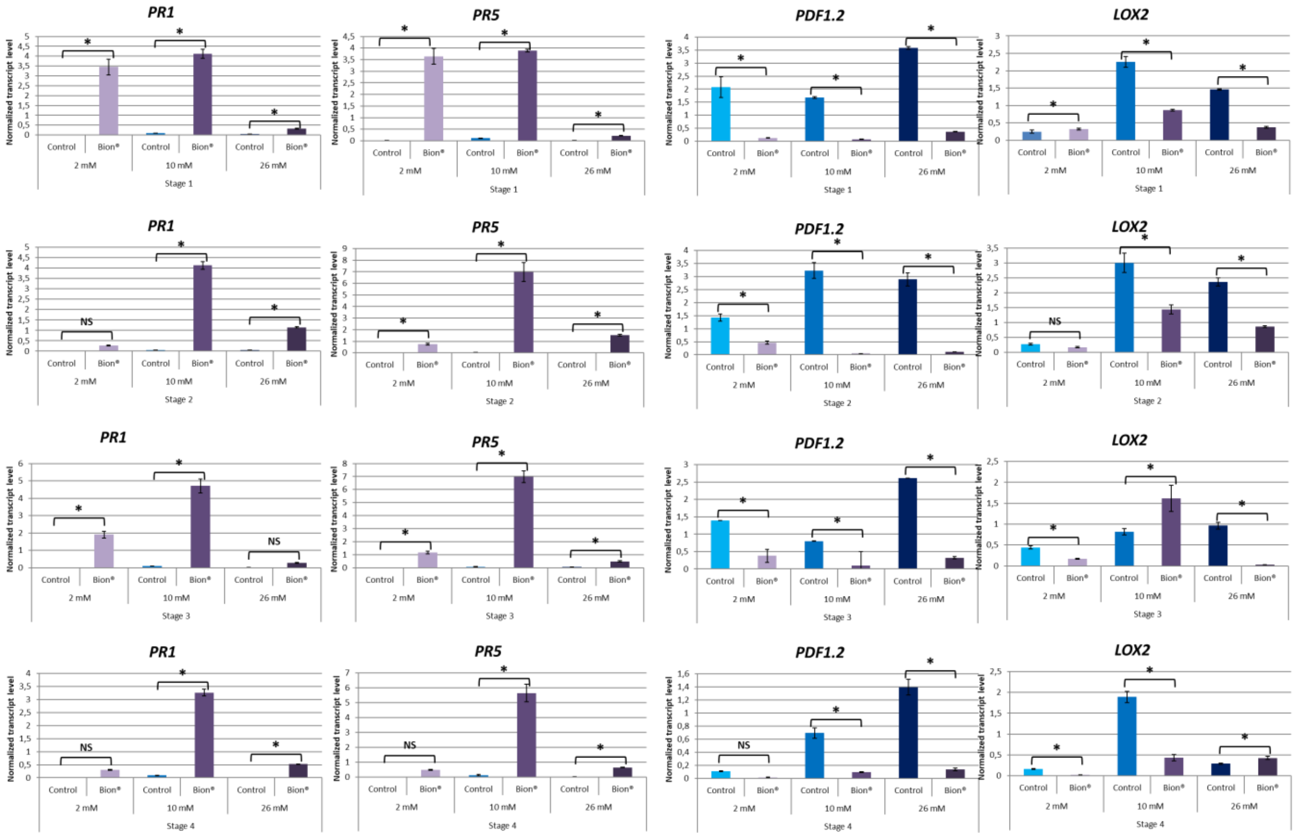

Supplementary Figure 2: Impact of nitrate nutrition on defense gene expression 48h after Bion treatment. Plants were cultivated until the indicated developmental stages (4 stages) under the indicated nutritional conditions. They were treated with Bion or water as a control then harvested 48 h after treatment. Defense gene expression was monitored by q-RT-PCR. Data represent normalized transcript levels using Clathrin as a reference gene. Experiments were performed 3 times with similar results. Representative data are shown. Error bars represent standard error. Stars represent statistically significant differences between elicitor treatments and control (*p<0.05* as calculated by t-test).

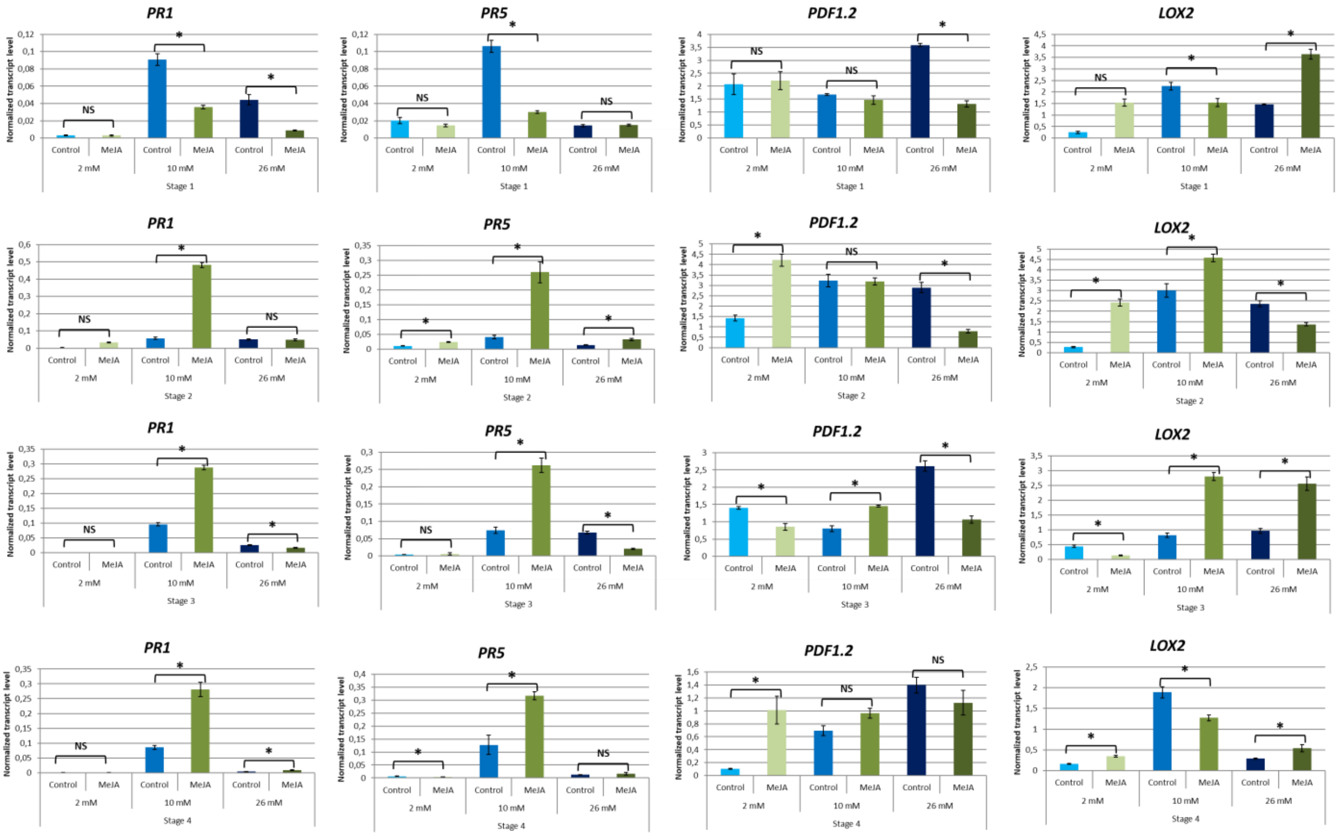

Supplementary Figure 3: Impact of nitrate nutrition on defense gene expression 48h after MeJA treatment. Plants were cultivated until the indicated developmental stages (4 stages) under the indicated nutritional conditions. They were treated with MeJA or water as a control then harvested 48 h after treatment. Defense gene expression was monitored by q-RT-PCR. Data represent normalized transcript levels using Clathrin as a reference gene. Experiments were performed 3 times with similar results. Representative data are shown. Error bars represent standard error. Stars represent statistically significant differences between elicitor treatments and control ($p<0.05$ as calculated by t-test).

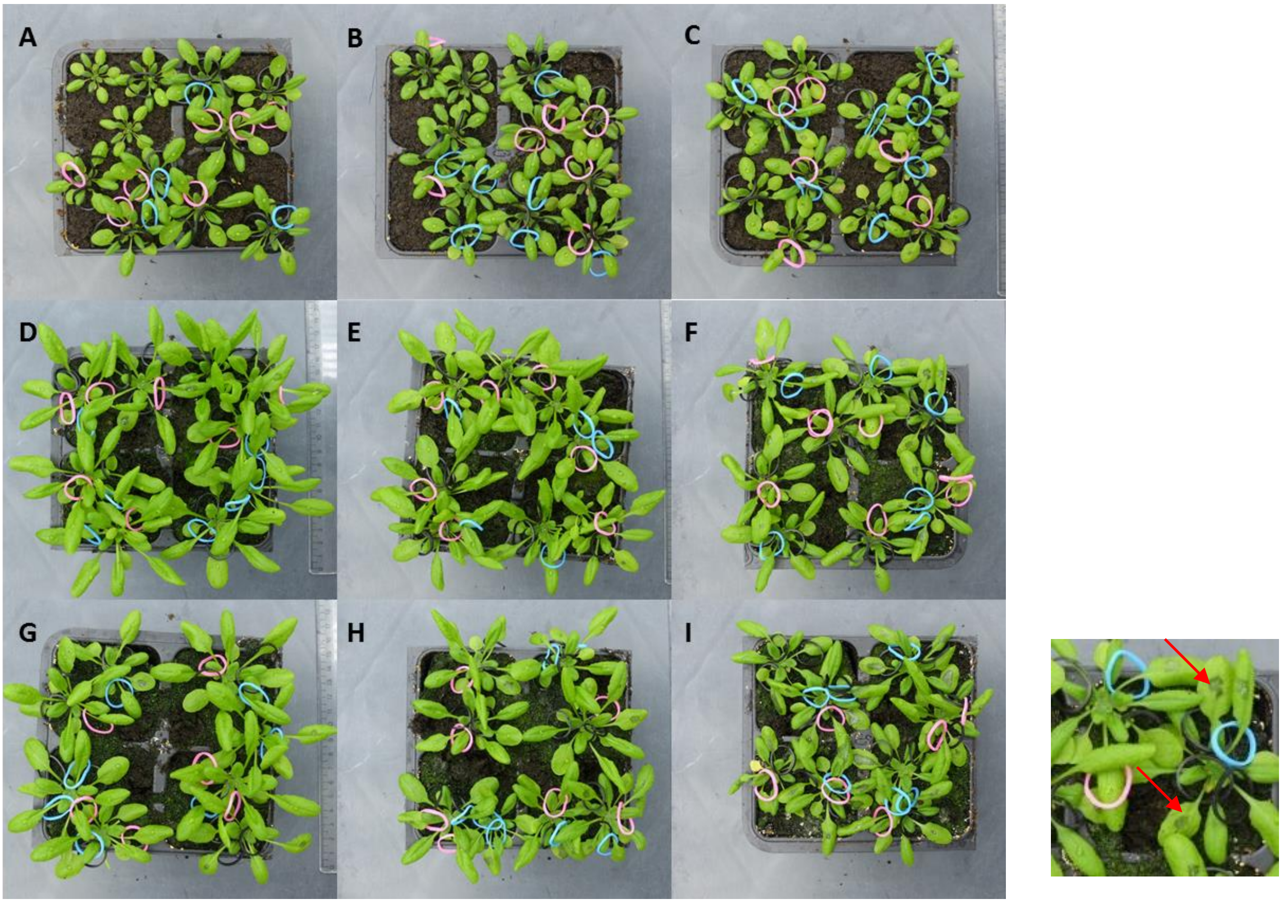

Supplementary Figure 4 : Symptoms of Arabidopis plants treated with plant defense stimulators and inoculated with *Dickeya dadantii*. Plants grown until stage 2 were treated with the indicated treatments (water as a control) then inoculated with *D. dadantii.* Pictures were taken 48h following infection with *D. dadantii*. **A**: 2 mM nitrate control, **B** : 2 mM nitrate Me-JA; **C :** 2mM nitrate Bion®; **D :** 10mM nitrate control, **E :** 10mM Me-JA, **F :** 10mM nitrate Bion®, **G :** 26mM nitrate control; **H :** 26mM nitrate Me-JA; **I :** 26mM nitrate Bion®. **K**: enlargement of a plant under 26 mM nitrate, control. Red arrows indicate symptoms

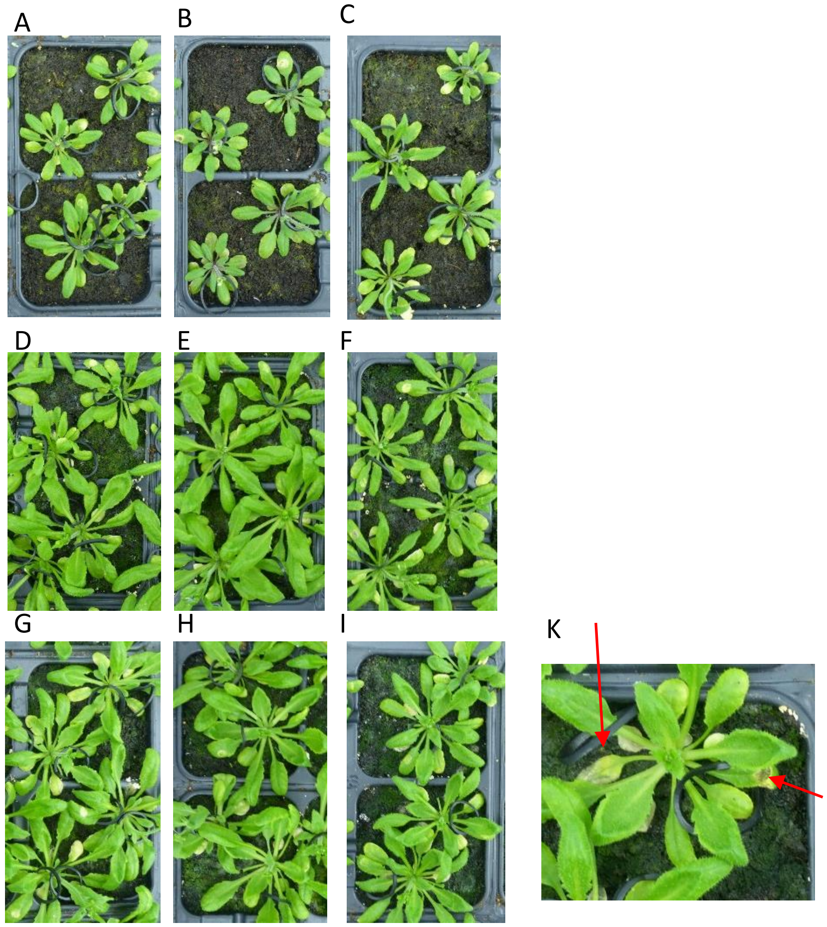

Supplementary Figure 5 : Symptoms of Arabidopsis plants treated with plant defense stimulators then inoculated with *P. syringae* pv. *tomato* DC3000. Plants grown until stage 2 were treated with the indicated treatments (water as a control) then inoculated with *P. syringae pv. tomato.* Pictures were taken 48h following bacterial inoculation. **A**: 2 mM nitrate control, **B** : 2 mM nitrate Me-JA; **C :** 2mM nitrate Bion®; **D :** 10mM nitrate control, **E :** 10mM Me-JA, **F :** 10mM nitrate Bion®, **G :** 26mM nitrate control; **H :** 26mM nitrate Me-JA; **I :** 26mM nitrate Bion®, **K**: enlargement of a plant under 26 mM nitrate, control. Red arrows indicate symptoms.
